# A New Approach for Improving Reliability of Personal Navigation Devices under Harsh GNSS Signal Conditions

**DOI:** 10.3390/s131115221

**Published:** 2013-11-07

**Authors:** Anup Dhital, Jared B. Bancroft, Gérard Lachapelle

**Affiliations:** Department of Geomatics Engineering, Schulich School of Engineering, The University of Calgary, 2500 University Drive NW, Calgary, AB T2N 1N4, Canada; E-Mails: adhital@ucalgary.ca (A.D.); jbancroft@gmail.com (J.B.B.)

**Keywords:** reliability, personal navigation devices, multipath, adaptive filter, Student's *t*-distribution, Variational Bayes, IMU, accelerometers, Doppler measurement

## Abstract

In natural and urban canyon environments, Global Navigation Satellite System (GNSS) signals suffer from various challenges such as signal multipath, limited or lack of signal availability and poor geometry. Inertial sensors are often employed to improve the solution continuity under poor GNSS signal quality and availability conditions. Various fault detection schemes have been proposed in the literature to detect and remove biased GNSS measurements to obtain a more reliable navigation solution. However, many of these methods are found to be sub-optimal and often lead to unavailability of reliability measures, mostly because of the improper characterization of the measurement errors. A robust filtering architecture is thus proposed which assumes a heavy-tailed distribution for the measurement errors. Moreover, the proposed filter is capable of adapting to the changing GNSS signal conditions such as when moving from open sky conditions to deep canyons. Results obtained by processing data collected in various GNSS challenged environments show that the proposed scheme provides a robust navigation solution without having to excessively reject usable measurements. The tests reported herein show improvements of nearly 15% and 80% for position accuracy and reliability, respectively, when applying the above approach.

## 1. Introduction

While personal navigation devices, including GNSS receivers and other self-contained sensors, are capable of providing highly reliable and accurate navigation solution in open sky environments, their performance still remains limited when it comes to navigating in GNSS-challenged environments such as natural and urban canyons. In such areas, GNSS signals, when available, are significantly affected by multipath effects and if used contribute to significant errors in the navigation solution while, if not used, result in lower solution availability. Moreover, GNSS suffers from other challenges, such as limited availability leading to poor geometry, high noise due to signal attenuation, non-normality of the measurement errors. For personal navigation systems that are implemented as an integrated Global Navigation Satellite System/Inertial Navigation System (GNSS/INS), especially those using relatively low cost micro electromechanical systems (MEMS) inertial measurement units (IMUs), the quality of GNSS signals plays a significant role in the navigation solution. The absence of GNSS or the presence of biased GNSS measurements can thus result in significant errors, depending upon the quality of the IMUs and the mechanization algorithm used.

The most common approach to address these challenges is to use a suitable fault detection and exclusion (FDE) scheme to identify and reject aberrant measurements. Receiver autonomous integrity monitoring (RAIM) is the most popular technique used for that purpose. There are numerous types of RAIM methods, based on implementation particularities. Nonetheless, all of these schemes are based on some kind of self-consistency checks among the available measurements. However, in harsh GNSS signal conditions such as in urban canyons, the FDE schemes are overwhelmed with various challenges like lack of sufficient measurement redundancy and simultaneous multiple faults. As GNSS measurements are already depleted in such environments, further rejection is not desirable since it limits the overall redundancy of the estimation and solution availability. Moreover, the measurement error distribution in those environments can no longer be assumed normal nor are the measurements uncorrelated in time, thus negating fundamental requirements of the standard Kalman filter.

Improper handling of faulty measurements can result in an unreliable navigation solution. Reliability is the level of trust that can be placed on the navigation solution provided by a personal navigation device. For example, if a navigation system estimates a position with accuracy (1σ) of 2 m along a particular axis, then it means that the error along that axis is expected to be less than 6 m with a confidence of 99.7% (*i.e.*, 3σ along that axis). However, if the true position along that axis is 10 m away from the estimated position, then the navigation solution is considered unreliable. Hence, a reliable navigation solution should have good consistency between the navigation solution and the estimated accuracy. Thus, in the context of this paper, reliability is defined as the capability of the navigation system to give a solution with proper accuracy and is formulated as the percentage of errors bounded by ±3σ or 99.7% (three times the estimated standard deviation of the position solution along a particular axis envelope).

In this regard, the main objective of this paper is to develop and analyze a new filtering algorithm for personal navigation systems that is more robust against outliers and optimizes the use of available GNSS measurements in harsh environments in order to obtain greater reliability. The paper identifies the significance of assumed GNSS measurement error distributions when determining the reliability of a navigation solution and proposes a method that assumes an adaptive error distribution which is more consistent with the true one. The method initially presented in [1] is further developed, tested and analysed in greater detail herein.

The proposed algorithm is detailed in the following section. Several field tests were used to validate the proposed algorithm. Section 3 describes how data were collected using a specific set of equipment in various environments. The results obtained by processing the data using the proposed algorithm are then analyzed in Section 4. Finally, Section 5 summarizes the main contribution of the paper and conclusions are drawn from the analyses in the previous section.

## 2. A Novel Filtering Strategy for Reliable Navigation

Any FDE scheme, by definition, is reliant on statistical tests that intrinsically require an *a priori* knowledge or assumption of the error statistics. In conventional FDE algorithms, the errors are often assumed to follow a normal distribution. Based on this assumption, a test statistics is computed in order to detect and remove unacceptably biased measurements, more specifically measurements that are statistically unlikely to occur.

When it comes to GNSS signal degraded environments, however, the error distributions tend to deviate from the assumed normal distribution thus deteriorating the performance of the traditional FDE schemes. The inconsistency between the assumed and the true error statistics often leads to removal of good measurements by the FDE or improper weighting of each measurement. Removing a good measurement in environments where GNSS is already depleted not only degrades the navigation solution but can also make the FDE scheme unavailable due to lack of sufficient redundancy.

In order to incorporate the GNSS measurements properly in the integrated navigation system, the FDE scheme should operate optimally. However, in harsh environments, FDE schemes face two major challenges, namely non-normality and varying error statistics. This paper, thus, attempts to address these challenges by adapting two approaches:

- Use of Student's t-distribution for GNSS measurement errors.
- Adaptation of GNSS covariance to the changing GNSS signal conditions.

### 2.1. Student's t-Distribution for GNSS Measurement Errors

In the Bayesian framework, the influence of measurement outliers on inferences for estimates, including population means and medians, can be reduced by replacing the normal distribution model by a heavy tailed distribution. Such heavy tailed distributions allow for the possibility of high noise and possibly biased observations. These distributions treat observations far from the regression line as high variance observations, yielding results similar to those obtained by deweighting the outliers [2]. This, to a certain extent, avoids having to reject measurements because of incorrect error modeling. Moreover, in the case of GNSS measurement errors occurring in environments like urban canyons where a significant proportion of measurements could be affected by multipath, a heavy tailed distribution is likely to be more representative of the true errors than the normal distribution, thus leading to more precise test statistics and hence a better FDE.

The use of a heavy tailed distribution such as Student's *t*-distribution, or simply *t* distribution, however, makes the filtering problem intractable as the functional models (process and measurement model) that appear in the form of likelihood function and transition density in the Bayesian framework do not reduce to a closed form solution as in the case of normally distributed errors. Two techniques are possible to implement the filtering algorithm with such a distribution. One option is to use Monte Carlo techniques such as particle filters [3]. However, these techniques are computationally very demanding and are not preferred in most of the commercial products. Hence, a computationally effective option is chosen for the implementation of the filter with *t* distribution. This method uses Variational Bayes (VB) framework wherein the *t* distribution is approximated as a normal mixture. The VB algorithm, which follows from [4], introduces an auxiliary random variable λ and estimates certain parameters using fixed point iteration as depicted in Algorithm 1.



**Algorithm 1:** VB filter
begin
$m_{k}^{-}\leftarrow \int f(x_{k-1})N(x_{k-1}|m_{k-1},P_{k-1})dx_{k-1}$
$P_{k}^{-}\leftarrow \int \left(f(x_{k-1}\right)-m_{k}^{-}){\left(f(x_{k-1})-m_{k}^{-}\right)}^{T}N(x_{k-1}|m_{k-1},P_{k-1})dx_{k-1}+Q_{k}$*λ̅* ← 1 for k from 1 to M do
$\mu_{k}\leftarrow \int h(x_{k})N(x_{k}|m_{k}^{-},P_{k}^{-})dx_{k}$
$S_{k}\leftarrow \int (h(x_{k})-\mu_{k}){\left(h(x_{k})-\mu_{k}\right)}^{T}N(x_{k}|m_{k}^{-},P_{k}^{-})dx_{k}+\frac{1}{\lambda_{k}}R_{k}$
$C_{k}\leftarrow \int (x_{k}-m_{k}^{-}){\left(h(x_{k})-\mu_{k}\right)}^{T}N(x_{k}|m_{k}^{-},P_{k}^{-})dx_{k}$
$K_{k}\leftarrow C_{k}S_{k}^{-1}$
$m_{k}\leftarrow m_{k}^{-}+K_{k}(y_{k}-\mu_{k})$
$P_{k}\leftarrow P_{k}^{-}-K_{k}S_{k}K_{k}^{T}$*D_k_* ← ∫(*y_k_* − *h*(*x_k_*))(*y_k_* − *h*(*x_k_*))*^T^N*(*x_k_* ∣ *m_k_*, *P_k_*)*dx_k_*
${\bar{\gamma }}_{k}\leftarrow tr(D_{k}R_{k}^{-1})$
${\bar{\lambda }}_{k}\leftarrow \frac{\vartheta +d}{\vartheta +{\bar{\gamma }}_{k}}$ end forendwhere *f()* is the process model, *h()* is the measurement model, *Q_k_* is the process covariance matrix and *R_k_* is the measurement covariance matrix. Normal distribution parameters *m* and *P* are the mean and the covariance of the estimated state. The variable ϑ is the degree of freedom that determines the Kurtosis (heavy tailedness) of the *t* distribution. Finally, *M* is the number of VB fixed-point iterations.


For this work, the numerical integrations in the algorithm are computed using the Cubature rule [5]. Despite using the *t* distribution for measurement errors, some form of FDE is still needed in order to account for gross errors. Hence, a FDE technique similar to the one used for normal distributed measurement errors has been used. Due to the symmetry of the *t* distribution, the test statistic for a local test assumes the same form as for the normal distribution [6], namely:

(1) $$\xi =\frac{r_{i}}{\sqrt{{\left(C_{r}\right)}_{ii}}}.$$

The threshold, however, is derived from the *t* distribution, *Th*=*t*(*ϑ*, 1 − *α_t_*), where *α_t_* is the probability of false alarm for the *t* distribution and is commonly a design parameter.

### 2.2. Adaptation of GNSS Covariance to the Changing Signal Conditions

The navigation filters used in personal navigation devices usually make an *a priori* assumption of the measurement covariance of the sensors being used. For a relatively good MEMS IMU like Analog Device's ADIS16488 unit (Analog Devices, Inc., Norwood, MA, USA), a tactical grade MEMS IMU, the stochastic part of the measurement errors can be considered constant. However, the same may not apply for the case of GNSS measurements. For instance, the measurement covariance of non-line of sight (NLOS) GNSS measurements is most likely to increase as the user moves into a deep canyon. Hence, a fixed *a priori* covariance for GNSS measurements in harsh environments will not correctly represent the true error characteristics of GNSS measurements. Hence, some adaptive technique must be used to adaptively tune the measurement covariance of GNSS measurements. Several techniques have been proposed in the literature to adapt the GNSS measurement covariance to the changing signal conditions. These techniques however come with several drawbacks, including filter instability, divergence, computational complexity or poor accuracy [7,8]. Hence, in order to adapt the GNSS measurement variance with the changing signal conditions and considering the drawbacks of the prevalent adaptive techniques, a new technique initially proposed by [9] is used in this work. This method scales the *a priori* variance of the GNSS measurements before they are fed to the navigation filter and hence avoids various drawbacks of the earlier adaptive techniques.

**Figure 1.**
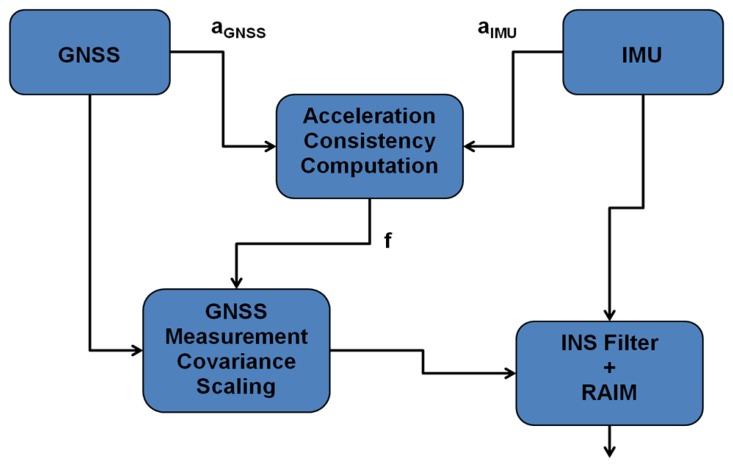
Block diagram of adaptive scheme.

The proposed method computes the user acceleration from GNSS Doppler measurements and compares its consistency with measurements from accelerometers in the IMU. With a tactical grade MEMS IMU, the user acceleration obtained using accelerometers is fairly accurate and hence is taken as a reference to compare with that obtained with GNSS Doppler measurements. Thus, during good GNSS signal conditions, the two accelerations are highly consistent whereas in GNSS signal degraded environments, the consistency between the two accelerations degrades. Based on this characteristic, a scaling factor is computed to scale the covariance of GNSS measurements thus adapting it in accordance with the changing GNSS signal conditions. A top level block diagram of the proposed method is depicted in Figure 1.

**Figure 2.**
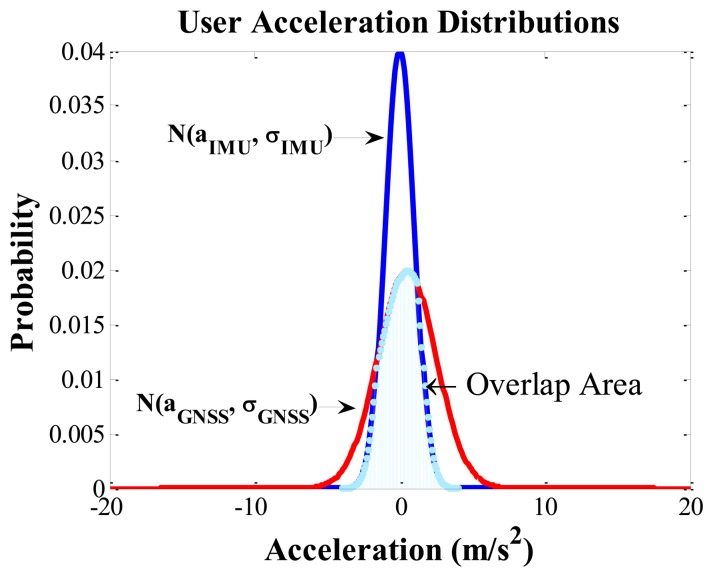
Consistency between accelerations.

Firstly, the user accelerations are computed along three axes of the Earth Centered Earth Fixed (ECEF) frame using the GNSS Doppler measurements. The details on the computation of user acceleration from GNSS are given in Appendix A. The user accelerations are also detected by the accelerometers inside the IMU. These accelerations, however, are in the IMU sensor frame. Thus they are transformed into the ECEF frame using rotation matrices so that they can be directly compared with the accelerations obtained using GNSS measurements. The errors associated with each solution are assumed to have a normal distribution. In the case when the GNSS measurement errors are assumed to have *t* distributed errors, the GNSS variance adaptive part, however, still assumes normal distribution for Doppler measurements while computing user acceleration from Doppler measurements. If *a_IMU_* and *a_GNSS_* are the acceleration values along a certain axis obtained using the accelerometer and GNSS respectively with associated standard deviations σ*_IMU_* and σ*_GNSS_*, then the consistency between the two accelerations can be expressed as the area overlap between the two acceleration distributions as shown in Figure 2.

The overlap between the distributions can be calculated as a Bhattacharyya coefficient (BC) [10]. Firstly, the Bhattacharyya distance (DB) for two univariate normal acceleration distributions is given as:

(2) $$BD=\frac{1}{4}\frac{{\left(a_{\text{IMU}}-a_{\text{GNSS}}\right)}^{2}}{\sigma_{\text{IMU}}^{2}+\sigma_{\text{GNSS}}^{2}}+\frac{1}{2}\text{log}\;\left(\frac{\sigma_{\text{IMU}}^{2}+\sigma_{\text{GNSS}}^{2}}{2\sqrt{\sigma_{\text{IMU}}^{2}\sigma_{\text{GNSS}}^{2}}}\right)$$

Then, *BC* is obtained as:

(3) $$BC=e^{-BD}*100\%$$

**Figure 3.**
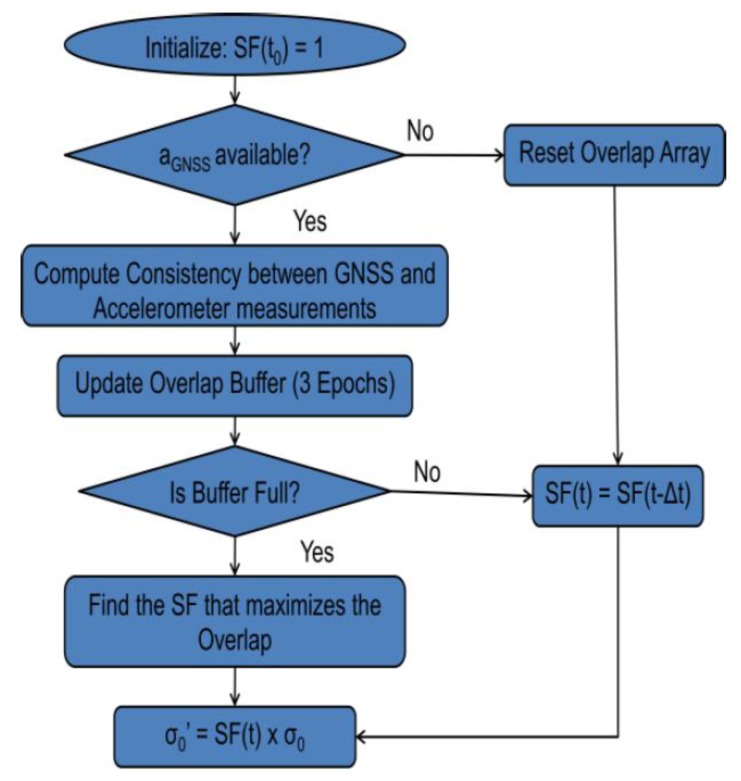
GNSS *a priori* variance computation.

Generally, in open sky conditions, the consistency between these two accelerations is high. However, in areas where the GNSS measurement quality degrades, the consistency between the two accelerations decreases since the errors in the GNSS derived acceleration distributions are no longer centered on zero. Thus, in areas with a good consistency, a higher weight (lower variance) is assigned to GNSS measurement updates while in areas with poor consistency, a lower weight is used. Then a scale factor based on the consistency between the accelerations is computed, which in turn is used to scale the *a priori* variance as shown in the block diagram of Figure 1. Figure 3 shows the proposed algorithm flowchart to select an adaptive *a priori* variance for GNSS measurements based on the consistency of the acceleration values obtained using the two sensors.

As with other non-adaptive filters, initially an assumption is made about the *a priori* variance for the GNSS measurements. The goal of the above algorithm is then to find a value to scale this *a priori* variance so that the assumed measurement noise distribution closely matches the true error characteristics. As mentioned in Appendix A, the condition of the minimum number of Doppler measurements required for obtaining the user acceleration must however be met before the consistency between the accelerations can be computed. The method described in Appendix A can be considered a loosely coupled consistency check as the comparison is done after the user accelerations are made available by the two systems. However, the comparison could also be done using a tightly coupled approach. With the knowledge of the orientation of the IMU and the direction cosines of the satellites, the accelerations on the IMU could be mapped to the line of sight acceleration from the Doppler derivative of each satellite. This method would obviate the condition of having a certain minimum number of Doppler measurements.

In order to mitigate the effect of noise in the Doppler measurements, the consistency is computed as a moving average of the consistency values at three GNSS epochs, each two typically separated by 50 ms. A buffer to store the consistency values of three consecutive epochs is thus maintained. This introduces a latency of two epochs at the beginning before the *a priori* variance scale factor can be computed. Moreover, if enough Doppler measurements are not available somewhere during the processing, then the buffer is reset as it has to hold the consistency values of three consecutive epochs.

Once the buffer is full, the current value of consistency is repeatedly computed by scaling the initial *a priori* variance with several *a priori* variance scale factors. The final *a priori* variance is chosen such that the moving average of the consistency values is maximized.

## 3. Equipment and Testing

**Figure 4.**
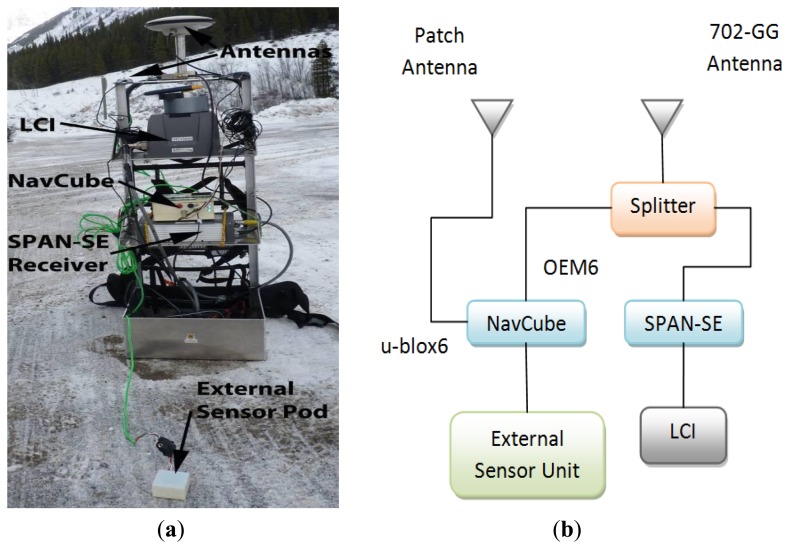
(**a**) Data collection equipment setup. (**b**) Block diagram of equipment setup.

The proposed scheme was evaluated by collecting pedestrian data in GNSS signal challenged environments. The data collection equipment setup depicted in Figure 4a was used for two scenarios. Data was collected with the NavCube, a data collection platform developed in the PLAN group of the University of Calgary [11]. The device includes four GNSS receivers, namely a NovAtel OEM628 (NovAtel Inc., Calgary, AB, Canada), an u-blox 6T (U-blox, Thalwil, Zurich, Switzerland), a SiRF IV (SiRF Technology, Inc., San Jose, CA, USA) and a Teseo II (STMicroelectronics, Geneva, Switzerland). It also includes an internal inertial sensor and the option to add other sensor sets through external cabling. All of the data is time tagged using the GPS time obtained from one of the receivers inside the device. The inertial data for the tests in this work were collected using an Analog Device's ADIS16488 sensor unit; related accelerometer and gyroscope specifications are given in Table 1.

A reference solution was also obtained for each experimental scenario in order to evaluate the performance of the proposed scheme. The reference system consisted of a NovAtel OEMV3 receiver as a base station at a pre-surveyed location. The rover part consisted of a NovAtel SPAN-SE receiver and a tactical grade LCI IMU. The reference solution was obtained as a tightly-coupled GNSS/INS solution computed using NovAtel's Waypoint Inertial Explorer post-processing software. The accuracy of the reference trajectory was better than 0.2 m.

As shown in the block diagram of Figure 4b, GNSS data collected using NovAtel's high performance GPS-702-GG antenna was split to feed the OEM6 (OEM628) receiver inside the NavCube and the SPAN-SE receiver. A small ANN-MS-0-005 patch antenna was used to collect GNSS data for the high sensitivity u-blox6 (u-blox 6T) receiver inside the NavCube. Inertial data was collected using the ADIS16488 external sensor unit, mounted besides the patch antenna on top of the backpack shown in Figure 4a. Pedestrian data was collected in two scenarios in order to thoroughly assess the proposed algorithms. The data collection environments are discussed in the following sub-sections.

### 3.1. Pedestrian Data in Urban Canyon

**Figure 5.**
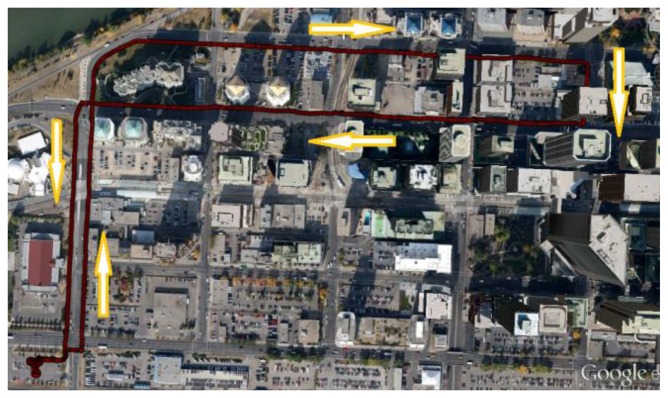
Reference trajectory (urban canyon).

The canyon created by the presence of tall buildings in either side of a street makes the navigation very challenging, especially for navigation systems with GNSS as a major component. The presence of multiple NLOS multipath signals with significant biases and limited visibility of the satellites degrades the quality and availability of the GNSS measurements. Hence, in order to assess the proposed scheme in GNSS challenged environments, pedestrian data was collected in downtown Calgary, Canada. The test environment presented an elevation mask angle varying from about 15 to 75 degrees. The test duration lasted over 40 min. The reference solution obtained for the urban canyon test is shown in Figure 5.

### 3.2. Pedestrian Data in Natural Canyon

**Figure 6.**
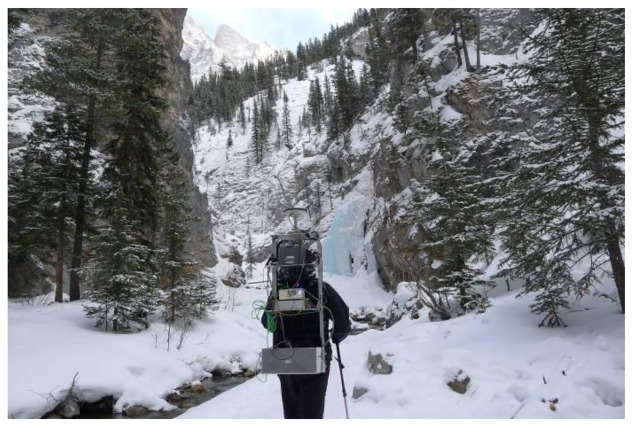
Data collection environment (natural canyon).

Unlike urban canyons where multipath is more specular, the multipath in natural canyons is generally more diffuse. The variation in the type of multipath while still limiting geometry and availability provides a unique test of the algorithms presented herein. Using a similar equipment setup as the urban scenario, data was thus collected in a natural canyon (King's Creek Canyon) in Kananaskis Country, AB, Canada. GNSS data collected using two receivers, namely OEM6 and u-blox6, were separately integrated with the IMU data collected using the ADIS16488 sensor unit. The test environment is shown in Figure 6.

**Figure 7.**
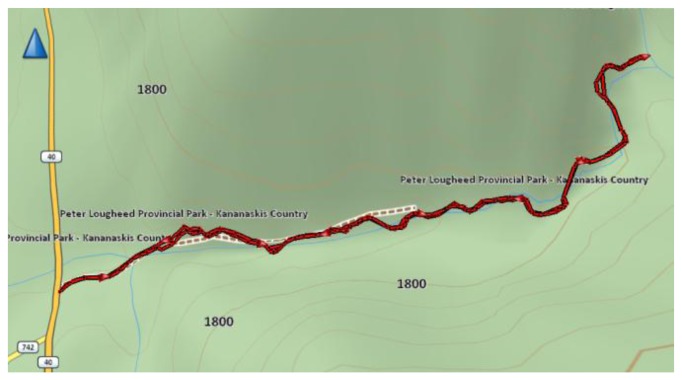
Reference trajectory (natural canyon).

The satellite mask angles in this environment varied between 50 and 80 degrees. The test duration was two hours. The reference trajectory obtained for the natural canyon data set is shown in Figure 7. The trajectory starts at Highway 40 and follows the creek until its end, 1,500 m later. The same snow path was easy to follow exactly on the way back. The fact that the same path was followed was followed forth and back was also used to assess trajectory repeatability. However, despite following the same path in both directions, the error characteristics and signal availability does not remain the same due to continuous changes in satellite geometry.

## 4. Results and Analyses

Prior to testing the proposed algorithm for an integrated navigation system, the suitability of using a heavy tailed distribution was examined through an initial test carried out using only GNSS range and Doppler measurements. GNSS data was collected in pedestrian mode under fairly open sky conditions. The OEM6 receiver inside the NavCube along with the GPS-702-GG antenna was used to collect the data. Simulated errors were added to three satellite measurements for nearly 70% of the data in order to analyze the performance of the filters in presence of known faults. The simulated errors on the three satellites consisted of uniformly distributed pseudorange (ρ) errors ranging from 10–70 m and uniformly distributed Doppler (Ø̇) errors ranging from −10 Hz to 10 Hz (±1.9 m/s). The results obtained with the navigation filter implemented with the assumption of *t* distributed measurement errors were compared against the results obtained with the assumption of normally distributed measurement errors. The comparisons were done for three cases, namely: (i) measurements without simulated errors; (ii) measurements with simulated pseudorange errors and (iii) measurements with simulated pseudorange as well as Doppler errors. Root mean square errors (RMSE) as well as reliability were calculated in local coordinates as shown in Table 2. It can be seen that the filter with the *t* distribution for measurement errors are least affected by the faults. It is also noted that, for the case with no added errors, the results are analogous for the two filter types. In fact, for an open sky data similar to the one used in this particular test, GNSS measurement errors follow the normal (N) distribution more closely as compared to the *t* distribution. This is supported by the slightly better horizontal accuracy as well as better reliability along the local axes in case of the filter that assumes normal distribution for the measurement errors.

Taking the above result as an affirmation of the suitability of the *t* distribution for GNSS challenged environments, the VB filter was further implemented in the GNSS/INS integrated system. The GNSS/INS filter itself was realized using the tight-coupling technique implemented in a C++ software application [12]. The proposed algorithm was finally assessed by analyzing the results obtained by processing the two pedestrian data sets described earlier.

### 4.1. Pedestrian Data in Urban Canyon

**Figure 8.**
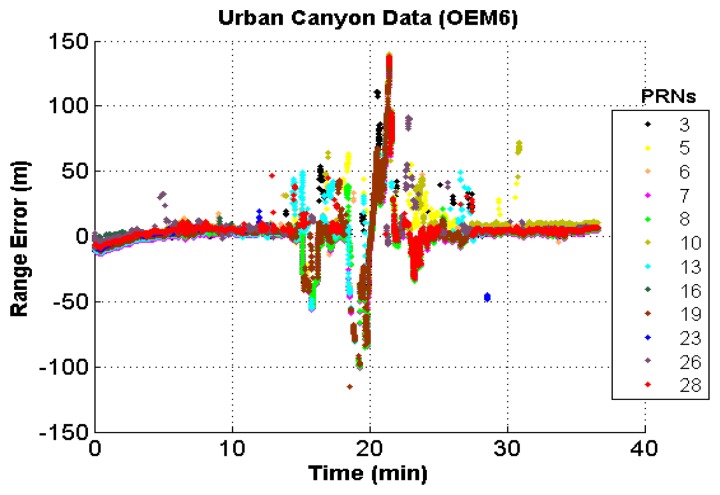
Range errors in urban canyon (OEM6).

As discussed in Section 3, GNSS data in harsh environments such as urban and natural canyons are significantly affected by multipath. A rough idea about these measurement discrepancies would be very helpful on analyzing the results. In this regard, the approximate range errors of all the GNSS measurements were computed using the procedure described in Appendix B. The range errors thus obtained for the OEM6 receiver in the urban canyon is depicted in Figure 8 as a function of time.

It can be observed that the range errors are quite significant at many epochs, exceeding well over 100 m. The presence of NLOS multipath signals and signal fading can cause the receiver to generate such erroneous measurements through shift of the NCO and distortion of the correlation function.

**Figure 9.**
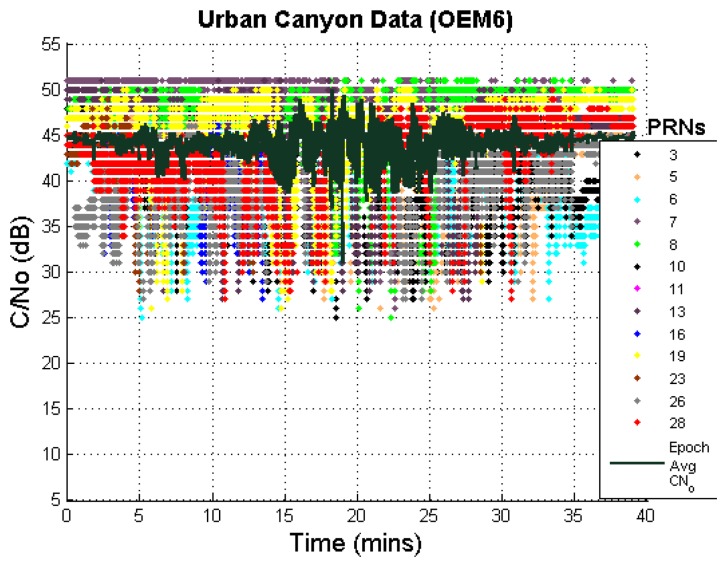
C/N_o_ values of measurements in urban canyon (OEM6).

Additionally, the carrier to noise ratios (C/N_o_) of available GNSS measurements are plotted in Figure 9 and show low values for many measurements in the middle section of the test duration. Such low values will result in higher measurement noise. This further shows the limitations of signal conditions in the test environment.

**Figure 10.**
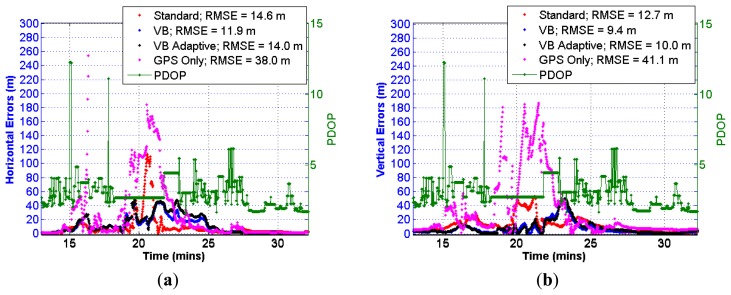
(**a**) Horizontal errors (OEM6 + IMU)—urban canyon. (**b**) Vertical errors (OEM6 + IMU)—urban canyon.

To assess the performance of the proposed scheme in such environment, a standard tightly coupled GNSS/INS filter with residual based FDE was first implemented whereby the GNSS range and Doppler measurement errors were assumed to follow a normal distribution. The GNSS measurement distribution was then replaced by the *t* distribution thus transitioning the navigation filter to a VB filter. Finally, the variance adaptive scheme discussed in Section 2.2 was also implemented along with the VB filter. These three cases are termed as: (i) *Standard*; (ii) *VB* and (iii) *VB Adaptive*, respectively. Since C/N_o_ based weighting has been found to be more robust in harsh GNSS signal conditions, the Sigma-ε variance model discussed by [13] was used to scale the GNSS range and Doppler measurements in all three cases. The performances of these three filters were first inter-compared by computing absolute values of horizontal and vertical errors. These horizontal and vertical errors are plotted only for the portion of the data in which the GNSS conditions were very harsh and the errors were more severe as depicted in Figure 10a,b. However, it is noted that the error values can grow very large corresponding to the times for which the satellite geometry was very poor as indicated by the dilution of precision. Thus, to segregate the effect of biased and diffused measurements from that of poor satellite geometry, the errors statistics were computed only for the times in which the position dilution of precision (PDOP) values were less than or equal to 10. The effect of masking in the urban environment is clearly depicted by the high PDOP values plotted alongside the errors in Figure 10a,b. Moreover, the portion of the data in which the user was static in a relatively open sky condition with many line of sight (LOS) GNSS measurements was also discarded in order to confine the analysis to degraded environments.

It can be observed from the above figures that the errors are smaller in the case of VB filters thus indicating the robustness of using the *t* distribution in environments with multipath laden GNSS measurements. The maximum horizontal error decreases by a factor of about 2.5 when using VB and VB Adaptive as compared to the standard filter. The accuracy seems to slightly degrade in the adaptive case. This is most likely due to subtle over-bounding of the assumed GNSS error distribution. Such over-bounding decreases the weight of the GNSS measurements. This de-weighting often leads to the dilution of unaffected measurements, thus degrading accuracy. However, the adaptive scheme leads to a highly reliable navigation solution as will be presented below. The above figures also include error plots for the GNSS only solution which was realized using an extended Kalman filter (EKF) that assumes a normal distribution for range and Doppler measurements. A standard residual based FDE was also implemented to detect and eliminate aberrant measurements. The error plots for the GNSS only solution gives an idea about the GNSS signal conditions in the test environment. It is observed from the above figures that there are few instances where the errors are in the range of around 200 m, thus leading to much higher RMSE as compared to the integrated GNSS/INS solutions.

**Figure 11.**
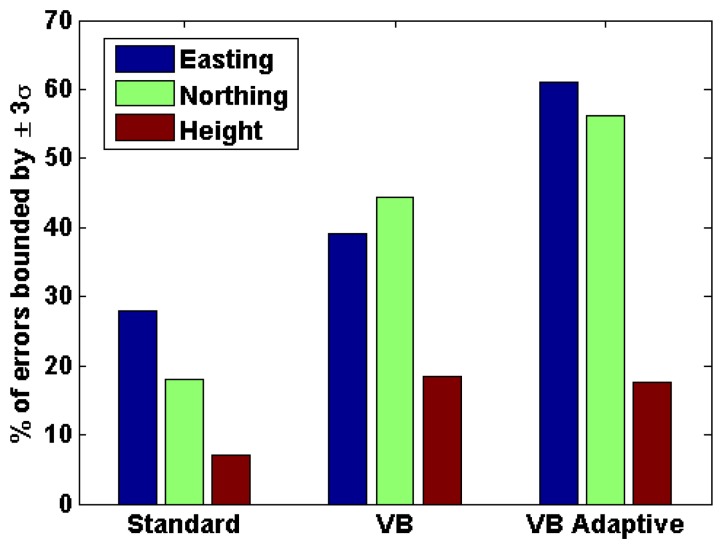
Reliability of position solution (OEM6+IMU)—urban canyon.

As discussed in Section 1, one of the most critical parameters for many applications using personal navigation devices is the reliability of the navigation solution. In this regard, the reliability of the navigation solution along the axes of the local plane for the three cases discussed above were computed as shown in Figure 11. It is observed that the reliability values are low for the standard filter. This is a common problem with standard Kalman filters, especially in integrated systems like GPS/INS, wherein the estimated accuracy of the navigation solution is often optimistic. However, the reliability values are found to improve with the VB filter and even further with the adaptive VB filter thus mitigating the problem of standard Kalman filters. These reliability results further indicate that, among the three filters, the adaptive VB filter best characterizes the true GNSS noise characteristics.

Availability of GNSS measurements is another key parameter to be assessed in such signal deprived environment. Since the *t* distribution inherently tends to retain more measurements, the availability of GNSS measurements was found to increase when using the *t* distribution. The percentage of range measurements rejected by the FDE decreased from 0.78% to 0.51% when going from the standard filter to the adaptive VB filter. The standard approach identified 18% of the Doppler measurements as faults and subsequently rejected them. This indicates that the Doppler measurements had an optimistic variance associated with the observation in comparison to the internal solution. This statistic pointedly shows how the standard method is insufficient since the system is incapable of handling the increased variance in the degraded environment. The VB filter alternatively rejected only 0.26% of the Doppler measurements. If the personal navigation system is also required to provide GNSS integrity parameters such as horizontal and vertical integrity limit, then a certain minimum requirement in terms of GNSS measurement availability must be met. When using only GPS, as in this work, a minimum of 5 GPS measurements must be available. However, in signal challenged environments, the available GNSS measurements are often less than five due to signal masking. Moreover, improper assumption of measurement noise statistics can also add to this unavailability. The availability of the integrity information for the three filters discussed above is tabulated in Table 3. Since there are only a few epochs with PDOP higher than 10, the exclusion of those epochs did not change the availability values by more than 0.1%. Thus the tabulated availability values were computed without discarding those epochs.

It can be observed that there is a slight improvement in terms of availability of GNSS integrity information with the VB filters as compared to the standard filter. The improvement could very well be magnified for a user navigating through GNSS challenged environments for a longer duration of time.

### 4.2. Pedestrian Data in Natural Canyon

As discussed in Section 3.2, the natural canyon data set includes GNSS data collected using two different receivers, namely an OEM6 and u-blox6. The data collected using these two receivers were integrated separately with the IMU in order to obtain the integrated filters for the three cases: Standard, VB and VB Adaptive filter. Using data from different GNSS receivers further validates the analysis of the performance of the proposed algorithm.

**Figure 12.**
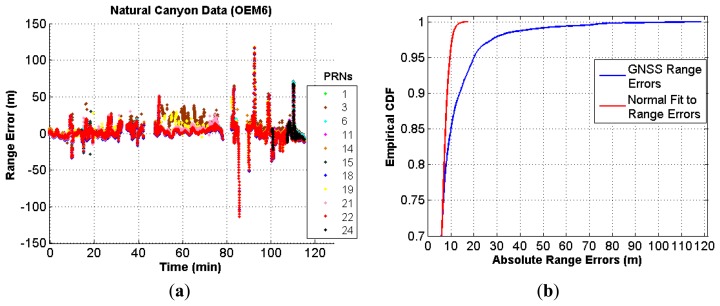
(**a**) Range errors in natural canyon (OEM6) (**b**) Range error distribution in natural canyon (OEM6).

Firstly, as with the urban canyon data, the range errors were computed using a similar technique as that for the urban data using the GNSS data from the OEM6 receiver. These range errors plotted in Figure 12a show that a significant portion of the data is affected by biased measurements, which at some epochs go as high as nearly 120 m.

Moreover, a cumulative density function (CDF) of absolute values of the range errors was also plotted along with the CDF of absolute values of the normal fit to the range errors. It can be observed in Figure 12b that there are not only errors with significant magnitude but there is also a high non-conformity between the two CDFs. This indicates that assuming a normal distribution for such errors is likely to lead to a sub-optimal navigation solution.

**Figure 13.**
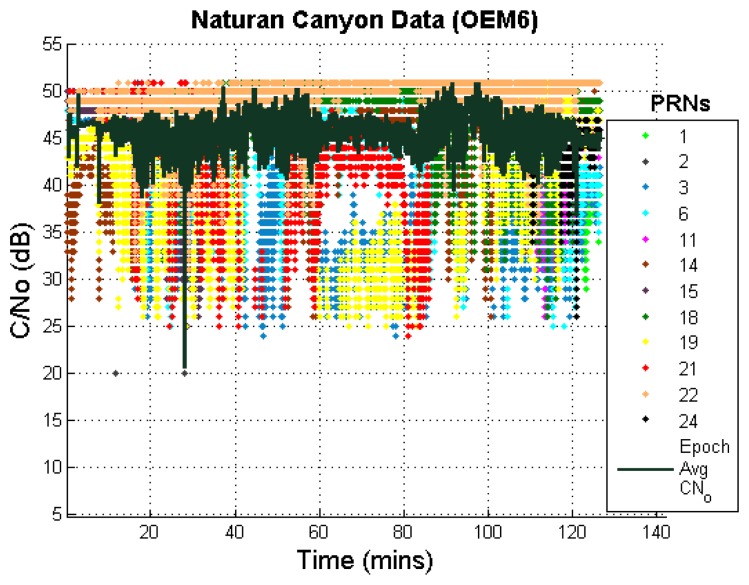
C/N_o_ values of measurements in natural canyon (OEM6).

The C/N_o_ values are plotted in Figure 13; these values are often below 40 dB. The position errors were computed as described earlier, neglecting the static LOS data at the beginning and end of the canyon. Moreover, the data with very high PDOP values (PDOP > 10) were also discarded during the position error computation.

**Figure 14.**
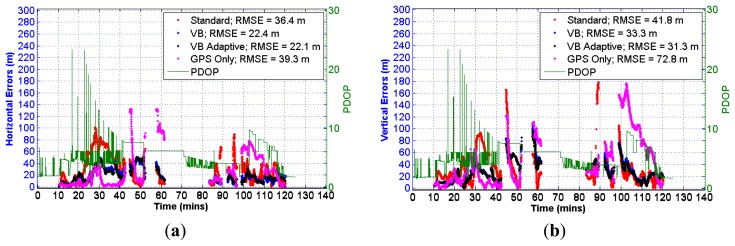
(**a**) Horizontal errors (u-blox6 + IMU)—natural canyon (**b**) Vertical errors (u-blox6 + IMU)—natural canyon.

The horizontal and vertical errors obtained using GNSS data from the u-blox6 receiver integrated with the IMU data are shown in Figure 14a,b. As before, the error values obtained for GPS are also included along with the PDOP values.

**Figure 15.**
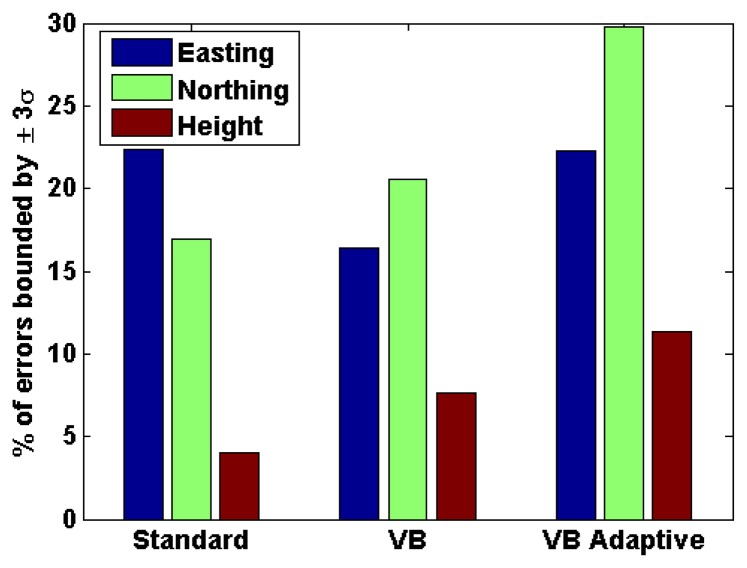
Reliability of the position solution (u-blox6 + IMU)—natural canyon.

It is observed that there is a significant improvement in accuracy using the proposed scheme as compared to the standard filter. The maximum errors are also found to decrease dramatically with the proposed scheme. The reliability values for the same data set, as depicted in Figure 15, again shows that there is an improvement in the reliability of the navigation solution with the proposed adaptive VB filter.

The absolute values of position errors and reliability values were re-calculated by processing the GNSS data collected using OEM6 receiver tightly integrated with the IMU data. The obtained results are tabulated in Table 4.

In this data set, although the accuracy improves only slightly, there is a significant improvement in reliability with the proposed scheme. This again indicates proper characterization of GNSS measurement errors through the use of adaptive *t* distribution for the GNSS measurement errors.

As for the urban canyon data, the availability of integrity information was also computed for the pedestrian data collected in natural canyon. The results tabulated in Table 5 indicate improvement in the availability of integrity information on using the proposed scheme.

## 5. Conclusions

A novel scheme that assumes a *t* distribution for GNSS measurement errors with an adaptable distribution variance to suit the changing signal conditions was presented. The proposed scheme was tested using realistic adverse environments GNSS signals. The results have shown that the proposed scheme is more robust as compared to the standard filter. The accuracy of the navigation solution was found to improve, although the improvement was not always significant. The reliability of the navigation solution, however, was significantly improved with the proposed scheme. Moreover, as this scheme mostly de-weights the bad measurements instead of removing them, the availability of GNSS measurements was also found to increase. The robustness of the proposed scheme validates the claim that replacing the assumption of normally distributed GNSS measurement errors by *t* distribution and changing its covariance using the presented technique to adapt to the changing signal conditions makes the assumed GNSS measurement noise statistics closer to the true statistics thereby making the proposed scheme highly effective for GNSS degraded environments.

## Figures and Tables

**Table 1.** IMU Specifications [11].

	**Parameter**	**ADIS16488**
**Gyroscopes**	In-Run Bias Stability (1σ)	6.25°/h
	Angular Random Walk (1σ)	0.3°/√h
	Rate Noise Density	23.8°/h/√Hz RMS

**Accelerometers**	In-Run Bias Stability (1σ)	0.1 mg
	Velocity Random Walk (1σ)	0.029 m/s/√h
	Noise Density	0.067 mg/√Hz RMS

**Table 2.** RMSE and reliability for open sky data.

			**No Errors Added**	**Errors Added on ρ**	**Errors Added on ρand** Ø̇
**RMSE (m)**	**N**	**Hor**	3.6	9.0	9.2
		**Vert**	2.3	7.9	7.5
	***t***	**Hor**	3.8	7.0	8.5
		**Vert**	2.3	6.9	6.3

**Reliability (%)**	**N**	**East**	93.4	73.0	68.4
		**North**	81.0	56.2	46.0
		**Up**	97.0	66.5	61.6
	***t***	**East**	72.3	71.9	70.0
		**North**	80.0	60.3	73.4
		**Up**	69.0	55.7	62.1

**Table 3.** Availability of GNSS integrity information (OEM6)—urban canyon.

	**Standard**	**VB**	**VB Adaptive**
**% of Epochs with Integrity Information available**	78.3	80.1	79.5

**Table 4.** RMSE and reliability (OEM6 + IMU)—natural canyon.

	**Absolute Position Errors (m)**		**Reliability (%)**		

	**Hor**	**Vert**	**East**	**North**	**Height**
**Standard**	17.2	12.1	32.7	32.5	36.7
**VB**	23.1	12.1	56.7	50.3	38.9
**VB Adaptive**	16.9	11.4	57.7	50.9	42.4

**Table 5.** Availability of GNSS integrity information—natural canyon.

		**Standard**	**VB**	**VB Adaptive**
**% of Epochs with Integrity**	**OEM6**	71.9	72.0	71.9
**Information Available**	**u-blox6**	65	74.5	74.5

## Appendix A Computation of User Acceleration from GNSS Measurements

The user accelerations can be obtained from GNSS using either twice differentiated phase measurements or single differentiated Doppler measurements. Due to the robustness of frequency lock loop in harsh environments, the Doppler measurements were used during this work. It follows from [14,15] that the differentiated Doppler observations (*Ḋ_i_*) are related to the user acceleration as:

(A1) $$\lambda_{i}{\dot{D}}_{i}=u_{i}(a_{rx}-a_{\text{sat}}^{i})+\frac{1}{\rho_{i}}\left[{\left|v_{\text{sat}}^{i}-v_{rx}\right|}^{2}-{\dot{\rho }}_{i}^{2}\right]+c\ddot{d}t+\varepsilon$$

where *u_i_* is the unit vector along the i^th^ satellite; *a_rx_* and *v_rx_* are the user acceleration and velocity vectors, respectively; $a_{\text{sat}}^{i}$ and $v_{\text{sat}}^{i}$ denote acceleration and velocity of the i^th^ satellite; *ρ_i_*, *ρ̇_i_* and *λ_i_* are the pseudorange, pseudorange rate and wavelength of the i^th^ satellite measurement, and *cd¨t* is the rate of change of clock drift. *Ḋ_i_* is the rate of change of Doppler and *ε* is the total error associated with the differential Doppler measurement. The instantaneous rate of change of the Doppler is obtained as:

(A2) $${\dot{D}}_{i}(t)=\frac{D_{i}(t+\;\Delta \;t)-D_{i}(t-t\;\Delta \;t)}{2\;\Delta \;t}$$

where *D_i_*(*t*+ Δ *t*) is the i^th^ Doppler measurement at time *t* + Δ *t* and *D_i_*(*t* − Δ *t*) is the i^th^ Doppler measurement at time *t* − Δ *t*. Thus, in a real time implementation, there is a latency of one GNSS epoch.

Using above equations, the user acceleration can be obtained using least-squares as:

(A3) $$\left[\begin{array}{c} \begin{array}{c} \lambda_{1}{\dot{D}}_{1}+u_{1}a_{\text{sat}}^{1}-\frac{1}{\rho_{1}}\left[{\left|v_{\text{sat}}^{1}-v_{rx}\right|}^{2}-{\dot{\rho }}_{1}^{2}\right]\\ \vdots \end{array}\\ \begin{array}{c} \vdots \\ \lambda_{N}{\dot{D}}_{N}+u_{N}a_{\text{sat}}^{N}-\frac{1}{\rho_{N}}\left[{\left|v_{\text{sat}}^{N}-v_{rx}\right|}^{2}-{\dot{\rho }}_{N}^{2}\right] \end{array} \end{array}\right]=\left[\begin{array}{cc} \begin{array}{cc} u_{x,1} & u_{y,1}\\ \vdots & \vdots \end{array} & \begin{array}{cc} u_{z,1} & 1\\ \vdots & \vdots \end{array}\\ \begin{array}{cc} \vdots & \vdots \\ u_{x,N} & u_{y,N} \end{array} & \begin{array}{cc} \vdots & \vdots \\ u_{z,N} & 1 \end{array} \end{array}\right]*\left[\begin{array}{c} \begin{array}{c} a_{rx,x}\\ a_{rx,y} \end{array}\\ \begin{array}{c} a_{rx,z}\\ \ddot{\text{cdt}} \end{array} \end{array}\right]+\left[\begin{array}{c} \varepsilon_{1}\\ \vdots \\ \begin{array}{c} \vdots \\ \varepsilon_{N} \end{array} \end{array}\right]$$

The use of least-squares requires a minimum of four Doppler observations (or five if GLONASS is also used in addition to GPS) to be able to use the proposed scheme and thus imposes a limitation.

## Appendix B. GNSS Range Error Computation

GNSS pseudorange measurements can be expressed as:

(B1) ρ=r+cdt+ε

where *ρ* is the pseudorange, *r* is the true range (true distance between the satellite and the receiver position), *cdt* is the receiver clock bias and *ε* is the total error which include atmospheric errors, orbital errors and multipath.

With the availability of an accurate reference solution for the user locations and with the knowledge of satellite positions from the ephemeris data, the true range *r* can be calculated. Now the problem lies in the segregation of *ε* from *cdt*.

The clock bias can be estimated in a filter by fixing the known receiver position and velocity from the reference solution and by modeling *ε* as a stochastic parameter. However, despite having a high observability obtained by fixing user position and velocity, accurate clock estimation cannot still be obtained. This is because of the fact that, in presence of large biases, *cdt* and *ε* get highly correlated due to which some part of *ε* gets consumed in *cdt* .

Thus, to mitigate the effect of multipath and other biases and noise in the estimation of *cdt*, an FIR filter is applied to the estimated clock bias. Finally, the range error is computed as:

(B2) $$\varepsilon =\rho -r-\hat{\text{cdt}}$$

where $\hat{\text{cdt}}$ is the filtered clock bias.
